# The effect of short pregnancy interval on perinatal outcomes in Turkey: A retrospective study

**DOI:** 10.12669/pjms.35.5.837

**Published:** 2019

**Authors:** Nevsen Saral, Seval Cambaz Ulas

**Affiliations:** 1 Nevsen Saral, Department of Midwife, Manisa City Hospital, Manisa, Turkey; 2 Seval Cambaz Ulas, Department of Midwifery, Faculty of Health Sciences, Manisa Celal Bayar University, Manisa, Turkey

**Keywords:** Low birth weight, Pregnancy interval, Preterm birth, Stillbirth

## Abstract

**Objective:**

The aim of the study was to determine the effect of short pregnancy interval on perinatal outcomes.

**Methods:**

The research was a retrospective study. The material consisted of birth records of a state hospital for the last three years in Manisa in the western region of Turkey (2015-2017) (n:8961). The research population included women whose gestational interval was ≤two years and the gestational week was over 22 weeks (n:2089). Perinatal outcomes were assessed through preterm birth, stillbirth, and low birth weight.

**Results:**

The mean age of women who are in the research group is 26.7 ± 5.32. According to the perinatal results of women with a pregnancy interval of two years and shorter; 8.2% of women had birth before 37 weeks and 0.3% resulted in stillbirth. It was determined that 4.8% of infants were born with low birth weight. There was no difference between the short pregnancy interval and stillbirth or preterm birth. However, a significant difference was found between the low birth weight and short pregnancy interval. (p>0.05).

**Conclusions:**

Pregnancy interval does not affect preterm birth and stillbirth from perinatal outcomes, but has a significant effect on the birth weight of the newborn.

## INTRODUCTION

The World Health Organization states that the pregnancy interval is time between the delivery date of the preceding live birth and conception date of the index pregnancy in women who have had more than one birth.^1^ The risk of complications increases in pregnancies that occur less than two years and they are considered to be high-risk pregnancies. High-risk pregnancies are of particular importance because of increased risk of illness or death before, during, and after birth.^2^ Nonoptimal pregnancy interval that is either too short or too long contributes to adverse maternal and perinatal outcomes in both low and high income countries.^3^ The other studies conducted on this issue support the results of this research. 4,5,6 Especially short pregnancy interval of less than 18 months have been associated with several bad fetal and neonatal outcomes such as pre-term birth, low birth weight (LBW), stillbirth, and newborn/infant mortality.^5^

Regardless of the gestational age, babies under 2500 grams of birth weight are considered “low birth weight”.^7^ The short period of time between the two pregnancies is reported to cause inadequacy of the renovation of the mother nutrient depot and consequently cause the baby to born with low birth weight.^8^ There is 22.8% chance of low birth weight in short pregnancy interval as compared normal pregnancy interval (12.1%).^9^

Short pregnancy interval is also a risk factor for preterm birth.^10^ Short pregnancy interval can cause preterm birth by increasing the risk of cervical insufficiency and infection.^11^ Preterm birth is prevalent up to 5% to 7% of among live birth in urbanized countries.^12^ It has been reported that the rate of preterm birth <37 weeks was higher in women with short pregnancy interval <12 (20.1%) compared with normal pregnancy interval (7.7%).^13^

The risk of neonatal death due to short pregnancy interval is high. The perinatal mortality was increased by 3-4 times in patients with an interval of fewer than 12 months between pregnancies, while infant mortality was increased by 2 times.^11^

Different cut-off points have been considered for nonoptimal pregnancy interval in the literature. The optimal pregnancy interval (24 months) was considered as the cut-off point in this study. The aim of this study was to determine the effect of short pregnancy intervals on perinatal outcomes.

## METHODS

The research is a retrospective study. The research used the birth records of a state hospital in Manisa in the western region of Turkey for the last three years (2015-2017) (N:8961). All pregnant women who met the criteria for including to the research in the years of 2015 – 2017 (n: 2089) were included. The criteria for including to the research are determined as gestational interval of the pregnant women should be ≤2 years and the gestational week of pregnant women should be over 22 weeks.

The data of the study were collected using a data collection form consisting of 21 questions. The data collection form was created by evaluating the data where the records that can be obtained through the system are complete. We evaluated three adverse perinatal outcomes: low birth weight (less than 2500 g), preterm birth (birth at less than 37 weeks’ gestation), and stillbirth. Number, percentage distribution and Chi-square test were used for the evaluation of research data. The study received approval from Medicine Health Sciences Ethics Committee of Manisa Celal Bayar University Faculty and state hospital.

## RESULTS

The time between two pregnancies in 55.0% of women in the research is shorter than 2 years. The mean age of women is 26.7 ± 5.32. The number of pregnancies for 44.4% of women was 2, and 52.9% had 2 live births. It was determined that 3.7% of the women in the research group had a chronic disease, and 9.8% were smoking (Table-I).

**Table I:** Descriptive characteristics of women by pregnancy interval.

Characteristic		Pregnancy Interval					

		2 years		<2 years		Total**	

		Number	%	Number	%	Number	%
Age	16-25	420	42.8	562	57.2	982	47.0
Mean ± Sd: (26.7±5.32) Min:16 Max:45	26-35	452	47.4	501	57.6	953	45.6
	36-45	68	44.2	86	55.8	154	7.4
Pregnancy number	2	435	46.9	493	53.1	928	44.4
	3	262	44.6	325	55.4	587	28.1
	4	132	43.9	169	56.1	301	14.4
	5 and above	111	40.7	162	59.3	273	13.1
Number of live births	1	34	15.3	188	84.7	222	10.6
	2	528	47.7	578	52.3	1106	52.9
	3 and above	378	49.7	383	50.3	761	36.5
The presence of chronic disease	Yes	30	39.0	47	61.0	77	3.7
	No	910	45.2	1102	54.8	2012	96.3
Smoking	Yes	91	44.4	114	55.6	205	9.8
	No	849	45.1	1035	54.9	1884	90.2

Total		940	45.0	1140	55.0	2089	100.0

** Column percentage is given.

The previous pregnancy of approximately 84.0% of women has resulted in a birth. The gestational age of birth for 0.8% of women (according to the ultrasound) is 33 weeks and below. It was determined that 1.1% of women had a pregnancy-induced disease and 36.7% had anemia (hemoglobin value was under 11.0 g/dl) (Table-II).

**Table II:** Pregnancy characteristics of women by pregnancy interval.

Characteristic		Pregnancy Interval					

		2 years		<2 years		Total***	

		Number	%	Number	%	Number	%
The shape of the previous pregnancy termination	Birth	872	49.8	880	50.2	1752	83.9
	Abortus	68	20.2	269	79.8	337	16.1
Gestational Age* (USG)	23-27	-	-	3	100.0	3	0.1
Mean±Sd (39.10±1.50)	28-33	4	28.6	10	71.4	14	0.7
Min:23.00. Max:42.60	34-39	637	45.9	750	54.1	1387	67.4
	40- 42	282	43.1	372	56.9	654	31.8
Pregnancy-Induced Disease	Evet	10	41.7	14	58.3	24	1.1
	Hayir	930	45.0	1135	55.0	2065	98.9
Hemoglobin value	7.20-9.00	51	44.0	65	56.0	116	5.6
Mean±Sd (11.54±1.52)	9.10-11.00	323	49.8	326	50.2	649	31.1
Min:7.20 Max:18.90	11.10-13.00	451	44.7	558	55.3	1009	48.3
	13.10 ve üzeri	115	36.5	200	63.5	315	15.1
Total		940	45.0	1140	55.0	2089	100.0

* 31 people have a missing data. ** Column percentage is given.

Looking at the characteristics of infants in the research; 99.7% were born alive, 99.5% of newborns were singular, 48.6% were female. The weight of 0.7% of infants are 2000g and below; 23.8% shorter than normal. The first minute of the APGAR score of 0.9% of the infant was between 0-6, and the fifth minute APGAR score of 0.4% of the infant was determined to be between 0-6. In addition, 8.7% of infants were determined to need intensive care (Table-III). The short gestational interval had no significant effect on preterm birth and stillbirth but it was determined to create a significant difference in terms of birth weight (Table-IV, p<0.05).

**Table III:** Descriptive properties of the newborn by pregnancy interval.

Characteristic		Pregnancy Interval					

		2 years		<2 years		Total**	

		Number	%	Number	%	Number	%
Number of babies	Singular	935	45.0	1144	55.0	2089	99.5
	Plural	5	50.0	5	50.0	10	0.5
Whether the baby is living	Live	942	45.0	1151	55.0	2093	99.7
	Dead	3	50.0	3	50.0	6	0.3
The gender of the baby	Female	420	41.4	597	58.6	1017	48.6
	Male	525	48.4	557	51.6	1082	51.4
Baby weight	2000g and below	7	35.7	9	64.3	16	0.7
	2010-2500g	27	30.0	56	70.0	83	3.8
	2510g and above	916	45.7	1084	54.3	2000	95.5
Baby’s length	47 cm and below	211	42.4	296	57.6	507	23.8
	48 cm and above	729	45.8	863	54.2	1592	76.2
Apgar score (1 min)	0-6	14	41.2	20	58.8	34	0.9
	7-10	931	45.1	1134	54.9	2065	99.1
Apgar score (5 min)	0-6	8	61.5	5	38.5	13	0.4
	7-10	937	44.9	1149	55.1	2086	99.6
Baby’s intensive care requirement	Yes	72	37.4	118	62.6	190	8.7
	No	870	45.7	1033	54.3	1903	91.3

Total		945	45.0	1154	55.0	2099	100.0

** The percentage of column is given.

**Table IV:** Evaluation of the effect of pregnancy interval on perinatal outcomes.

Characteristic		Pregnancy Interval						Test value*/p	

		2 years		<2 years		Total**			

		Number	%	Number	%	Number	%		
Preterm birth	<37 week	105	61.4	66	38.6	171	8.2	3.08	0.07
	≥37 week	1044	54.4	874	45.6	1918	91.8		
Stillbirth	Yes	3	50.0	3	50.0	6	0.3	0.06	0.80
	No	1146	55.0	937	45.0	2093	99.7		
Low Birth Weight (n:2099)	below2500g	65	69.1	29	30.9	94	4.5	7.95	0.00
	above 2500g	1084	54.3	911	45.7	1995	95.5		

Total		1149	55.0	940	45.0	2089	100.0		

* The Pearson Chi-squared test value,

** The percentage of column is given.

## DISCUSSION

The study showed that 55% of women become pregnant in less than two years. In a study of pregnancy interval results in Israel, it was reported that the pregnancy interval short than 24 months was 54.1%.^14^ In a prospective cohort study, 26.5% of the women included in the study had a pregnancy repeat within 18 months.^15^ It was found that this rate was 18.8% when the research data were re-evaluated according to gestation intervals of 18 months. The research findings are consistent with the relevant literature. 84.0% of women’s previous pregnancy resulted in birth. In a study where the effects of pregnancy interval were investigated, 89.5% of pregnancies resulted in birth.^16^

In the study, 8.2% of the births occurred before 37 weeks. It was found that the short pregnancy interval did not have a significant effect on preterm birth. The short pregnancy interval has been recognized as a risk factor for preterm birth.^17^ It was found that the short pregnancy interval is related with preterm birth rates according to the studies which were conducted in different countries as Tanzania and Canada.^18^,^19^ Contrary to this it was determined that short pregnancy intervals were not related with preterm birth rates in a study conducted in Pakistan which is similar to the findings of this study.^20^

The study also found that 0.3% of women had given stillbirth and the pregnancy interval did not have a significant effect on stillbirth. There are almost 3.2 stillbirths per 1000 births all over the world each year. The highest absolute numbers (approximately stillbirth rates are 32 per 1000) of stillbirths occur in Sub-Saharan Africa and South Asia.^21^ However ın many high-income countries (Europe, North America and Australia vb.), for every neonatal death there are now approximately 1.7 stillbirth (stillbirth rates are below 5 per 1000 births).^22^ The stillbirth rate in Turkey is 1 per 1000 births.^23^ The stillbirth rate of Turkey is on the level of developed countries. The stillbirth rate which was found in this study is lower than the rate for stillbirths of Turkey. The data for the research were acquired from the last three years’ data of a single hospital, therefore it supposed that there is not a significant relationship between stillbirth and short pregnancy interval.

It was determined that 4.8% of infants were born with low birth weight and that the short gestational interval created a significant difference in terms of baby’s birth weight. According to the data of the Turkish Population Health Survey (2013); 10% of the children whose birth weight are known are at a low birth rate. 23 Zhu et al determined that women with short pregnancy intervals had an increased risk of low birth weight in their babies.^24^ The findings of the researchs which were conducted in Egypt,^25^ Iran^26^ and Turkey^27^ have shown similar results. The present research findings are consistent with the literature.

## CONCLUSION

In this study, where the effect of short gestational intervals on perinatal outcomes is investigated, perinatal results were evaluated in three subheadings. Pregnancy interval does not affect preterm birth and stillbirth from perinatal outcomes but has a significant effect on the birth weight of the newborn. Furthermore, more than half of the women who were included to this study conceived before the optimal pregnancy interval in this study. It supposed that increasing to take proper contraceptive medicines and raising awareness of women for perinatal outcomes are important for both women and children health aspects.
